# New Chloroprene Rubber/Styrene–Butadiene Rubber (CR/SBR) Blends Cross-Linked with Tin(II) Oxide (SnO): Curing Characteristics, Swelling Studies, Mechanical Properties, and Flame Resistance

**DOI:** 10.3390/molecules29246028

**Published:** 2024-12-20

**Authors:** Aleksandra Smejda-Krzewicka, Konrad Mrozowski, Krzysztof Strzelec

**Affiliations:** Institute of Polymer and Dye Technology, Faculty of Chemistry, Lodz University of Technology, Stefanowskiego Street 16, 90-537 Lodz, Poland; krzysztof.strzelec@p.lodz.pl

**Keywords:** styrene–butadiene rubber, chloroprene rubber, tin(II) oxide, cross-linking, flame resistance, statistical analysis

## Abstract

This study aimed to investigate the properties of tin(II) oxide (SnO) as an unconventional cross-linking agent for chloroprene (CR) and styrene–butadiene (SBR) rubbers compositions. The use of tin(II) oxide results from the need to reduce the use of zinc oxide as a cross-linking agent due to environmental regulations and its toxic impact on aquatic environments. The studied elastomeric blends can be cross-linked with tin(II) oxide, and the results demonstrate the significant potential of this oxide in such applications. The CR/SBR vulcanizates cross-linked with SnO exhibit good mechanical properties and a high degree of cross-linking. The studies clearly show that the proportions of both rubbers as well as the amount of tin(II) oxide used influence the cross-linking of the CR/SBR blends and the properties of vulcanizates. FTIR spectrum analysis allowed the identification of the cross-linking mechanism, which followed the Friedel–Crafts alkylation reaction mechanism. The AFM analysis determined the miscibility of the rubbers and interelastomeric reactions, proving that the rubbers studied are partially miscible. The results of the oxygen index measurements indicated that the obtained vulcanizates showed flame resistance and self-extinguishing properties. Multivariate regression was performed to fit the models to the experimental value and to determine the influence of the content of the cross-linking agent and the CR and SBR proportions on the properties of the blends.

## 1. Introduction

Cured elastomers consist mainly of several or even more different compounds (ingredients). The rubber products only acquire valuable properties during the final processing cycle, i.e., cross-linking. The curing process creates a three-dimensional elastomer network, generating the characteristic elastic properties of these products. The elastic properties of rubber materials depend on the rubber type, the cross-linking agents, and the vulcanization conditions. During the cross-linking of rubbers, the chemistry of this process changes and this has important consequences on the network structure and properties [1,2,3,4,5]. The specific properties of cured rubber materials—high flexibility, the ability to undergo large elastic deformations during dynamic and static loads, resistance to chemical compounds, low permeability to water, liquids, and gasses, and good dielectric properties—ensure their wide application in various fields [4,5,6,7,8,9,10,11,12,13,14]. The rubber compounds are basic semi-products used to produce rubber goods. The type and amount of the ingredients incorporated into the rubber compound depend on the end application. Conditions and requirements in rubber products determine the type, quality, and quantity of ingredients used [1,14,15,16]. However, cross-linking elastomers are the most important part of the finishing process.

Vulcanization, i.e., the permanent, chemical, and transverse connection of rubber macromolecules, is possible thanks to chemical reactions and physical processes [17,18,19,20]. In this process, the type of elastomer and the type and amount of cross-linking agents are very important [17,20,21,22,23,24]. To cross-link rubber, functional groups with a specific chemical activity are necessary. The type of vulcanizing agent used depends on the chemical structure of the elastomer macromolecules. The effect of cross-linking and the main effect of elastomer deformation is the loss of solubility and the elimination of translational movements of macromolecules, leading to the elimination of the plastic deformation of the elastomer [23,24,25,26,27]. The increase in the degree of cross-linking changes all properties. The resilience, static and dynamic modulus, and hardness increase. The increase in the degree of network density leads to the growth of the tensile strength tear and fatigue resistance properties, which reach a maximum at a certain network density range [17,20,22]. In addition, the permanent deformation, friction coefficient, and hysteresis loss decrease [22,23,24,25,26,27].

The degree of elastomer cross-linking achieved as a result of vulcanization has a significant impact on the final properties of the vulcanizate as an engineering and construction material. The type and concentration of the resulting network nodes determine the mechanical and thermal properties of vulcanizates [18,19]. The elasticity change of the cured elastomer is the most important feature. It has been found that the elasticity increases linearly with the degree of cross-linking. In practice, the linear relationship occurs only in a certain range of the network density only, which is most likely related to the imperfections of the theory and network or the disintegration of macromolecules and cross-links, occurring simultaneously during the vulcanization [18,23,24,25,26,27].

With the increasing performance requirements applied to rubber materials, elastomer blends have become an object of considerable interest for researchers. This phenomenon has contributed to a deeper understanding of topics such as phase morphology and elastomer miscibility. Due to their properties, these blends are considered for applications such as coatings, adhesives, and automotive or aerospace parts [4,5,6,7,8,9,10,11,12]. Polymeric blends are defined as physical blends of at least two polymers [28,29,30,31,32]. Polymeric blending enables the production of new materials that can combine polymer properties that cannot be achieved using single-blend components [6,7,8,9,10,30,31,32,33,34,35,36,37,38,39]. Elastomeric blending methods are divided into mechanical and chemical methods [35,36,37,38,39]. The selected blending method and the type of polymers to be blended largely determine the level of homogeneity of the resulting polymer blend [25,35,36,37,39]. An important feature of an elastomer blend is its interfacial behavior and the possibility of interelastomer reactions, which can be divided into three groups [40]:

- Reactions between two elastomers activated by a low-molecular substance that is not involved in the formation of cross-links;
- Reactions between two elastomers activated by a low-molecular substance that participates in the formation of cross-links;
- Reactions without the participation of cross-linking substances.

In the first group, the cross-linking reactions of elastomers or elastomer blends with metal oxides can be observed [40,41,42,43,44,45]. This is due to the reactivity of the activated functional groups of macromolecules. A good example in this case would be the cross-linking of a CSM/SBR blend with zinc oxide. The cross-linking of such blends is carried out by the Friedel–Crafts alkylation of SBR phenyl rings by polyhalide. This is catalyzed by aprotic Lewis’s acid, which is ZnCl_2_. This is formed in situ from added initiators. In this case, the progress of the process and the properties of the resulting product may depend on the alkylation conditions, the structure and the proportion of CSM in the CSM/SBR blend, and the type of metal oxide application [46]. In addition to this composition, Estagy et al. [41], to improve the adhesion and miscibility of EPDM and SBR, used Lewis’s acid, AlCl_3_, to form an EPDM-g-SBR copolymer via Friedel–Crafts reactions.

Another group of reactions that occur in elastomer blends are activated by low-molecular substances involved in forming cross-links. This includes curing, as well as co-curing, where network knots are formed following the reactions of cross-linking substances with double bonds or α-methylene groups of macromolecules. Considering the poor thermodynamic miscibility in the case of elastomers, the sphere of interelastomeric reactions is usually not quite extensive and does not extend beyond the phase separation boundary [47,48,49,50,51].

Self-cross-linking blends are also a group of materials where interelastomeric bonds occur. In such types of blends (elastomer/elastomer or elastomer/plastomer), one of the elastomers can cross-link the other due to the ability of the reactive functional groups of one component to react with the functional groups of the other component in the absence of the cross-linking agent. The formation of network knots in a well-matched mix is observed, for example, when heating epoxidized natural rubber (ENR) with chloroprene rubber (CR), carboxylated acrylonitrile–butadiene rubber (XNBR), and chlorosulfonated polyethylene (CSM). Very often, an ester bond is involved in the formation of the cross-links. This is seen especially frequently in blends with XNBR [52,53,54,55].

**Figure 1:**
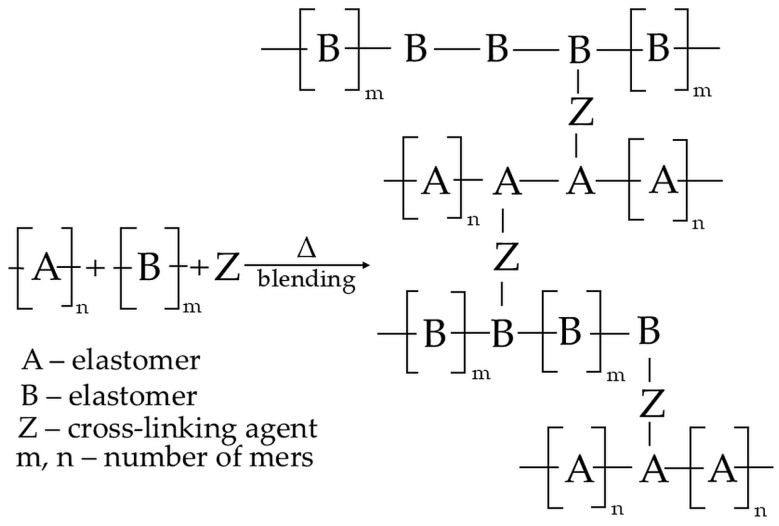
The schematic of an interelastomeric spatial network connected by Z cross-links [46].

In addition, it is still necessary to establish that the resulting reactions between macromolecules can occur intramolecularly in the same macromolecule or intermolecularly between different macromolecules. The first option results in a modification of the chain structure and, for example, a change in the degree of unsaturation, branching, or isomerization. On the contrary, the second option results in reactions between two different macromolecules to form an interelastomeric spatial network [40,56]. Figure 1 schematically visualizes the mechanisms behind interelastomeric reactions connected by cross-links.

The choice of such a pair significantly influences the properties of final rubber products created by combining two different elastomers. The price is also an important aspect so that the product’s overall value is not lost when mixing more expensive polymers with cheaper ones. This method also seeks to eliminate the individual defects of elastomers through such combinations and through the selection of suitable fillers to modify their properties [7,8,9,10,11,12,13,14].

This study aimed to characterize the effect of tin(II) oxide (SnO) on the curing of chloroprene and styrene–butadiene rubber (CR/SBR) blends and the properties of the products obtained. Based on infrared spectroscopy, we proposed the mechanism of interelastomeric bond formation between chloroprene rubber and styrene–butadiene rubber cured with tin(II) oxide. Additionally, the flame resistance and mechanical properties of CR/SBR/SnO products were tested.

## 2. Results and Discussion

### 2.1. Cross-Linking Mechanism of CR/SBR Composites in the Presence of Tin(II) Oxide

**Figure 2:**
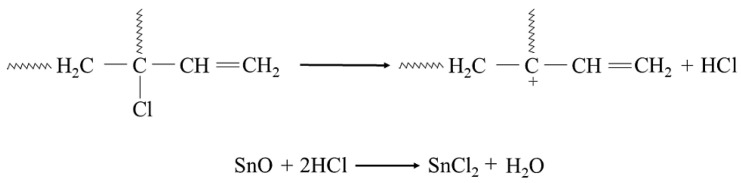
Dehydrohalogenation of 1,2-chloroprene mers of chloroprene rubber and formation of SnCl_2_.

In the study, it was found that during the heating of a blend containing chloroprene rubber and styrene–butadiene rubber in the presence of tin(II) oxide, interelastomeric bonds are formed between the investigated elastomers. It is worth mentioning that the phenyl group in the chain of styrene–butadiene rubber (SBR) is involved in the cross-linking with chloroprene rubber (CR). The chloroprene rubber is primarily composed of 1,4-trans-chloroprene and 1,4-cis-chloroprene units. The impact of an electro-negative chlorine atom bonding to a carbon atom at the double bond is so strong on the double bond that the 1,2-chloroprene units participate in CR cross-linking [57,58,59,60]. The cross-linking of CR/SBR blends in the presence of tin(II) oxide begins with the dehydrohalogenation of chloroprene rubber with hydrogen chloride (HCl) release. The hydrogen chloride reacts with the tin(II) oxide, leading to the formation of tin(II) chloride as Lewis acid (Figure 2), which works as an obligatory catalyst for bond formation between elastomers.

**Figure 3:**
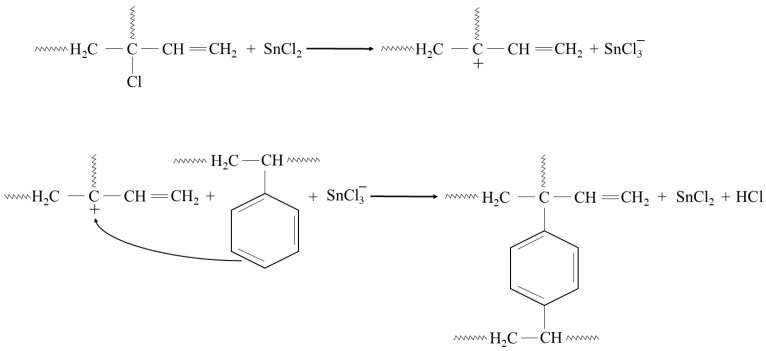
CR-SBR interelastomeric bonds formed due to the Friedel–Crafts alkylation of SBR phenyl rings by CR catalyzed with SnCl_2_.

**Figure 4:**
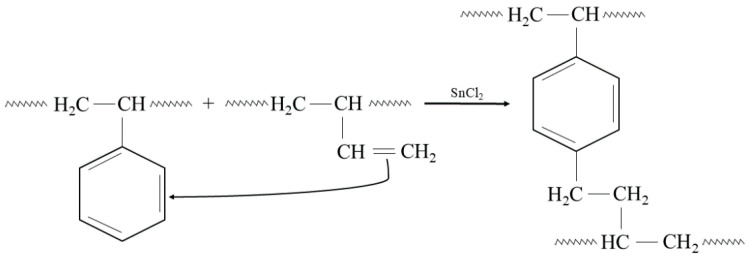
The autoalkylation of phenyl rings by SBR chains containing side vinyl groups, occurring in the presence of SnCl_2_ in the tested CR/SBR blends.

In addition, the presented cross-linking mechanism (Figure 3) shows that CR and SBR blends can be cross-linked with tin(II) oxide. The heating of SBR/CR/SnO compositions is coupled with the formation of SnCl_2_ generated in situ. It acts as a catalyst for the interelastomeric Friedel–Crafts alkylation of SBR phenyl rings by CR as an elastomeric polyhalide. The alkylation of the phenyl rings presumably applies analogously to blends in the mentioned research [42,43,44,45,59,60]. The autoalkylation of SBR phenyl rings by the vinyl side groups of SBR mers, catalyzed by SnCl_2_, is also possible in the CR/SBR blends (Figure 4) [57,58,59]. In this case, chloroprene rubber is the donor and SnCl_2_ is the acceptor of chloride ions.

### 2.2. Effect of Tin(II) Oxide on Structure and Dispersion of CR/SBR Blends

**Figure 5:**
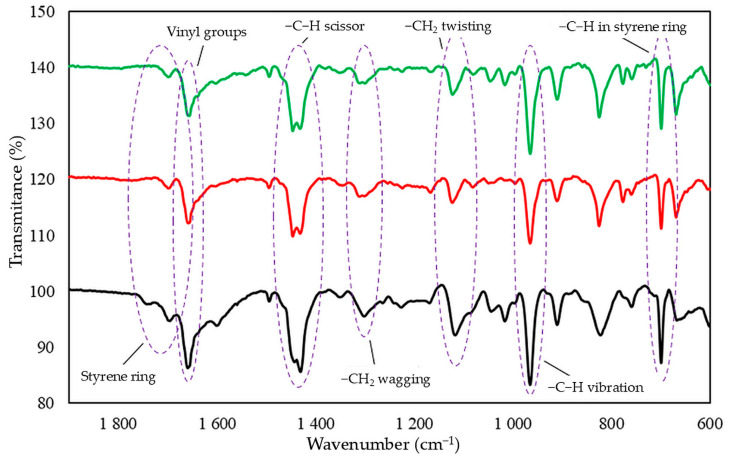
IR spectrum of uncross-linked CR/SBR (75/25 by wt.) blend as a standard (dark line), CR/SBR/SnO (75/25/1.8 by wt.) blend before (red line) and after (green line); cross-linked at T = 433 K; t = 30 min.

Infrared spectra confirmed that tin(II) oxide is capable of cross-linking CR/SBR blends (Figure 5). Heating of CR/SBR blends containing SnO leads to a decrease in the intensity of the absorption bands in IR at a wavenumber of 700 cm^−1^ (out-of-plane –C–H band in the SBR aromatic ring). The intensity of the absorption peaks decreased during the heating of CR/SBR blends, which may indicate changes in the degree of substitution of the phenyl ring. Furthermore, an increase in band intensity can be observed at a wave number of 964 cm^−1^ (–C–H– vibration in vinyl groups in 1,4-trans-butadiene mers). The absorption peaks at the wavenumbers of 1180 and 1300 cm^−1^, corresponding to the twisting and wagging of the –CH_2_ groups, respectively, show increased intensity. This may suggest the formation of bonds between the macromolecular elastomers, possibly due to the disappearance of double bonds in the CR chain. Additionally, there is an increased peak intensity at a wavelength of 1470 cm^−1^, corresponding to the C–H scissor vibrations in the elastomer chains. This could indicate a reorganization of structures when the blends were heated in the presence of SnO. Small intensity changes can also be observed in the range of 1670–1740 cm^−1^, characterizing differences in the degree of phenyl ring substitution. These observations confirm the alkylation of SBR phenyl rings by CR, catalyzed by SnCl_2_ generated in situ. Likewise, with the use of ZnO or Fe_2_O_3_ [44,45], autoalkylation reactions of phenyl rings with SBR chain fragments containing vinyl side groups occur in CR/SBR/SnO composites. The analysis of IR spectra allows us to assume that the cross-linking of chloroprene and styrene–butadiene rubber blends in the presence of tin(II) oxide occurs according to the Friedel–Crafts alkylation reaction mechanism. Tin(II) chloride (SnCl_2_), which is generated in situ as Lewis acid, acts as a catalyst for the reaction (Figure 3). The reaction takes place between styrene rings and carbocation (CR chain with a disconnected chloride ion).

**Figure 6:**
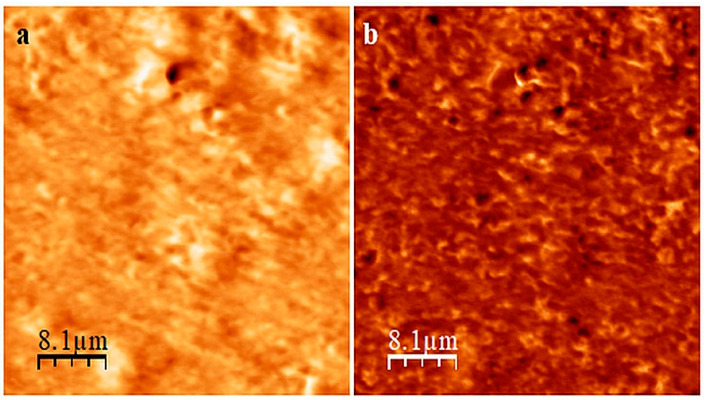
AFM images of CR/SBR/SnO blend (75/25/1.8 by wt.) before (**a**) and after (**b**) cross-linking at T = 433 K; t = 30 min.

The analysis of the AFM images of the CR/SBR (75/25 by wt.) composition before and after the cross-linking (Figure 6) confirms that curing the CR/SBR blend in the presence of SnO leads to an improved degree of SBR dispersion in the CR matrix. Before the cross-linking, it was evident that there were two phases, with signs of continuity in the CR/SBR/SnO compositions (Figure 6a). However, after cross-linking, the dispersion of SBR in the CR matrix appears more droplet-like (Figure 6b). This may indicate the occurrence of some interactions between chloroprene rubber and butadiene–styrene rubber in the studied blends cross-linked with tin(II) oxide.

**Figure 7:**
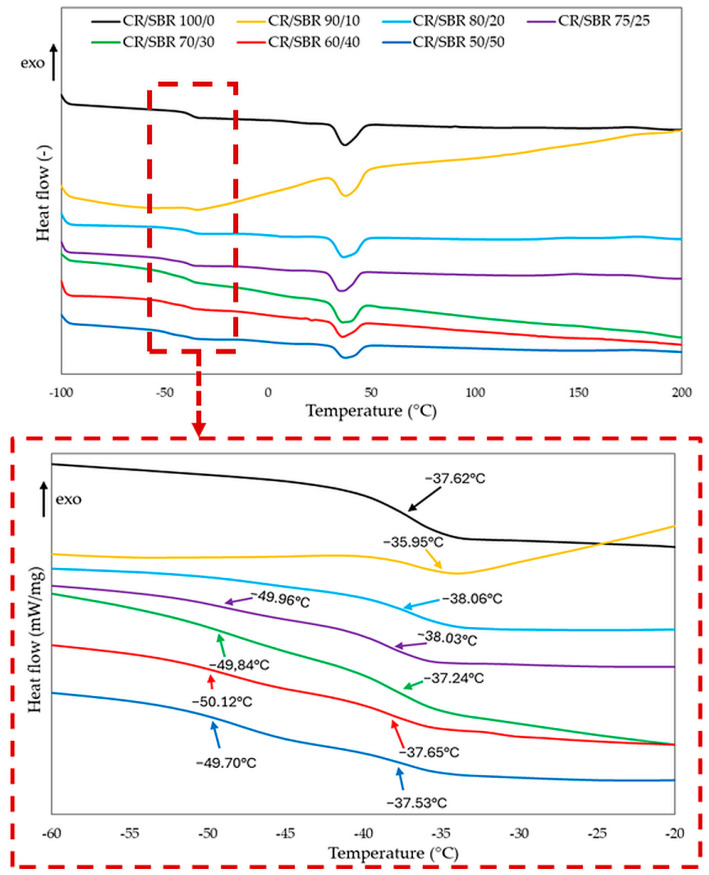
Differential scanning calorimetry (DSC) curves of CR/SBR blends cross-linked with tin(II) oxide.

The confirmation of the interelastomer reactions between chloroprene and butadiene–styrene rubbers in the presence of tin(II) oxide can be the differential scanning analysis. The interelastomer reactions occurring between CR and SBR can lead to the improvement of the homogeneity and miscibility of the tested compositions. It can be observed from the DSC curves (Figure 7, Table 1) that for the compositions containing from 50 to 75 phr of chloroprene rubber, two glass transition temperatures occurred, and each of them corresponded to the individual phases of the blend, i.e., CR and SBR. In these composites, the chloroprene rubber had a glass transition temperature in the range of −38.0 to −36.5 °C, while the second glass transition temperature in the range of −50.1 to −49.7 °C should be assigned to the butadiene–styrene rubber. However, the changes in the determined glass transition temperatures for the CR/SBR = 50/50, 60/40, 70/30, and 75/25 by wt. blends may indicate the presence of interactions and increased compatibility between SBR and CR under the influence of tin(II) oxide used as a curing agent. The DSC result was surprising for the CR/SBR = 80/20 by wt. and CR/SBR = 90/10 by wt. blends, for which only one glass transition temperature was observed (T_g_ = −38.0 °C, and T_g_ = −36.0 °C, respectively). One glass transition temperature may prove the complete miscibility of SBR with CR and the accompanying interelastomer reactions that are formed under the influence of tin(II) oxide used for cross-linking such blends.

It is worth noting that the DSC thermogram is the result of all reactions occurring in the selected temperature range. It should be remembered that many competing reactions occur during the heating of the elastomer composite; therefore, it is tough to measure the enthalpy for a specific response. In the DSC curves of all CR/SBR blends and compositions containing only chloroprene rubber, a distinct endothermic peak was found at about 37 °C, which corresponded to the crystalline phase of chloroprene rubber. This phenomenon is the typical behavior of CR, which can crystallize during storage or stretching and become amorphous during heating.

The DSC curves also show that the cross-linking of CR/SBR blends occurred in the temperature range from 125 °C to 243 °C (Table 1). Such a low cross-linking onset temperature most likely indicates the existence of a pre-curing process, to which CR is particularly susceptible. The enthalpy determined during cross-linking can be correlated with the degree of cross-linking. The higher the enthalpy value, the more effective the cross-linking of the blends. In the case of CR/SBR blends heated in the presence of tin(II) oxide, the exothermic peaks were broad and very low at comparable enthalpy values (ΔH = 1.78–5.79 J/g). The progress of cross-linking is mainly indicated by the type of rubber and the cross-linking agent used. In these studies, only low intensities of exothermic peaks were observed. This probably indicates the limited efficiency of tin(II) oxide as a cross-linking agent for the elastomer systems studied.

### 2.3. Effect of SnO on Cross-Linking and Properties of CR/SBR Blends

#### 2.3.1. Degree of Cross-Linking of CR/SBR Composites Determined by Vulcametric and Swelling Properties

The study of cross-linking kinetics among elastomeric materials is one of the main determinations required to perform the vulcanization process optimally. The cross-linking of rubber is associated with heat exchange, but also with chemical reactions occurring in the elastomeric medium, causing irreversible changes in the material’s properties. This process fundamentally alters the material’s mechanical, thermal, and chemical properties, making the rubber stronger, more elastic, and resistant to environmental factors like heat, chemicals, and deformation. Therefore, the study of cross-linking kinetics yields important parameters, such as the optimal vulcanization time (t_90_), scorch time (t_02_), increment of torque (ΔT), and minimum torque (T_M_). These properties determine the degree of cross-linking, the mix’s viscosity, the period of safe processing, and the time required to achieve optimal final product properties. Tin(II) oxide was chosen due to its ability to create aprotic Lewis acid and the need to reduce the use of zinc oxide in the rubber industry. The effect of CR/SBR ratios (0/100, 50/50, 60/40, 70/30, 75/25, 80/20, 90/10, or 100/0) on the cross-linking and properties of blends cured in the presence of SnO as the Sn^(2+)^ ion donor was initially investigated. Based on previous studies, we initially used 2.4 phr of SnO per 100 phr of CR.

**Figure 8:**
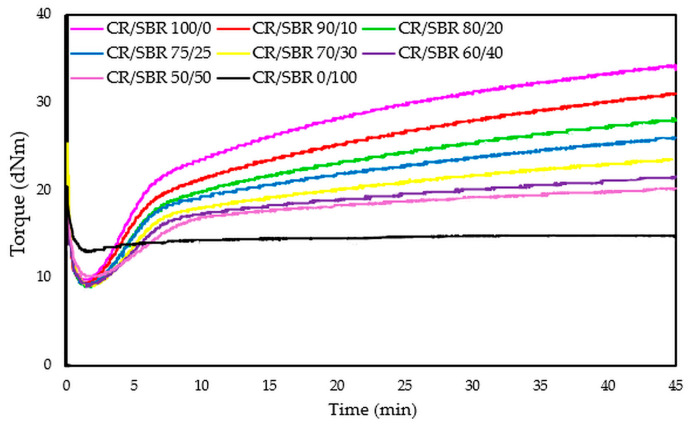
Rheometric curves of CR/SBR blends cross-linked with tin(II) oxide at 160 °C.

The compositions of the blends and the results of vulcametric properties, equilibrium swelling, and elasticity constants are summarized in Table 2. After analyzing the results, it was found that the cross-linking of the CR/SBR/SnO blends occurs without reversion, and the change in the rheometric torque is dependent on the amount of chloroprene rubber and rises with its proportion. The CR/SBR (90/10 by wt.) blend has the highest torque increment after 30 min of heating (ΔT_30_) among the composites, amounting to 18.7 dNm. The CR (100 by wt.) sample shows the highest torque increment (ΔT_30_ = 21.1 dNm) in general. It is worth mentioning that the CR rubber is responsible for the start of the cross-linking process, and so a larger amount allows for a greater degree of cross-linking and a higher torque increment. The course of rheometric kinetics for all CR/SBR blends using tin(II) oxide is presented by the rheometric curves in Figure 8.

**Figure 9:**
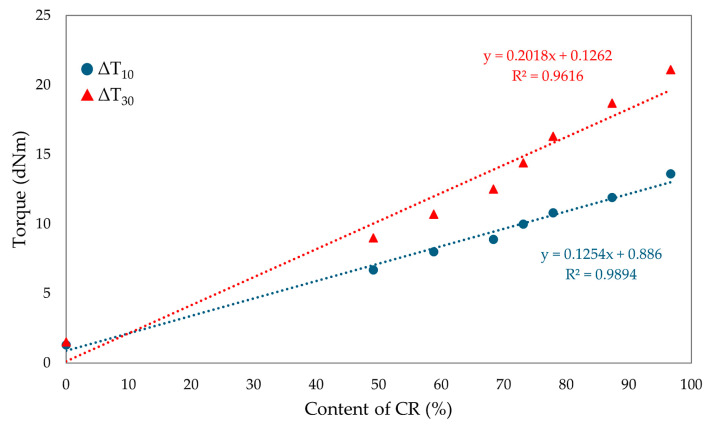
The effect of CR content in CR/SBR/SnO blends on the torque increment at T = 433 K.

The torque increment of blends heated in the presence of SnO is smaller compared to the values seen using a standard cross-linking agent such as ZnO (ΔT_30_ = 26.0 dNm for composites containing 100 phr of CR and 3 phr of ZnO) [61]. The differences in cross-linking rates between CR/SBR/ZnO and CR/SBR/SnO blends are probably due to the different activity of Sn^(2+)^ ions compared to Zn^(2+)^ and those of SnCl_2_ and ZnCl_2_, which are Lewis acids. It can also be noted that the scorch time does not show any trend; however, the incorporation of 50 phr of chloroprene rubber extends the t_02_ value to 2.5 min. Other results oscillate around 2 min. There was no significant effect of the amount of chloroprene rubber on the values of the scorch time of the tested compositions. Figure 9 shows the effect of the CR content in CR/SBR/SnO blends on the torque increment heating at T = 433 K. A linear trend was observed, where the higher the chloroprene rubber content, the higher the torque increment values. This was related to the formation of interelastomeric bonds.

**Figure 10:**
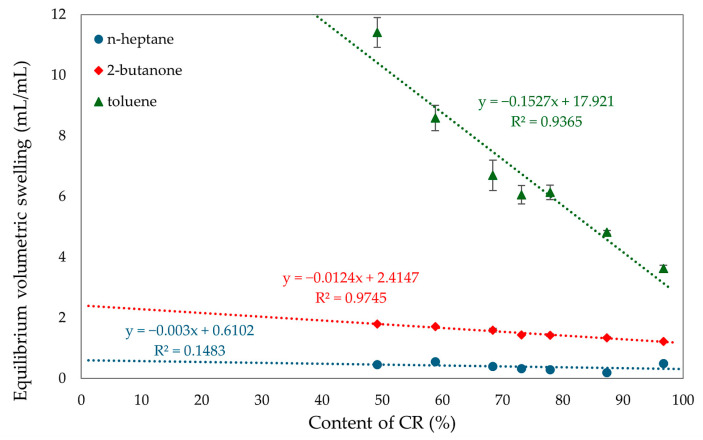
The effect of CR content on equilibrium swelling in various solvents of CR/SBR composites cross-linked with tin(II) oxide at T = 433 K; t = 30 min.

A higher amount of chloroprene rubber in the vulcanizates results in a higher degree of cross-linking, indicating the formation of more bonds during the cross-linking. A higher content of SBR rubber results in decreasing values of elasticity constants, this is also related to the fact that CR rubber is the initiator of vulcanization. The more CR the more bonds during the cross-linking process. The correlation between the degree of cross-linking and the proportion of rubber in the composites was confirmed by the equilibrium volume swelling results in toluene, 2-butanone, and n-heptane. The results, summarized in Table 2 and Figure 10, indicate that an increase in CR content means a higher degree of cross-linking and a lower Q_v_ value. The content of chloroprene rubber affects swelling in toluene, while for other solvents these values are comparable. The sample with SBR alone dissolved, and this was due to the inability to cross-link SnO of this rubber. The low value of equilibrium volume swelling indicates the formation of many cross-bonds. This value can be related to the degree of cross-linking. The CR/SBR (90/10 by wt.) vulcanizate obtained the lowest swelling value in all media. For toluene it was Q_v_^T^ = 4.82 mL/mL, for 2-butanone it was Q_v_^M^ = 1.35 mL/mL, and for n-heptane it was Q_v_^H^ = 0.20 mL/mL. This value indicates that the resistance of CR to n-heptane is related to the polar rubber being insoluble in a non-polar solvent. The opposite is true for non-polar SBR, which is soluble in heptane. The swelling results illustrate this relationship; as the SBR content in the composition increases, the Q_v_^H^ and Q_v_^T^ also increase. The analysis of the content of the eluted substance during equilibrium swelling shows the marked differences between the content of the eluted fraction determined experimentally. The theoretically calculated values confirm that, in the CR/SBR/SnO vulcanizates, a reaction occurs between CR and SBR macromolecules with the formation of an interelastomeric spatial network. The quantity of the eluted fraction during the swelling in toluene and n-heptane differs from the ratio between CR and SBR. With higher CR. content the amount of leached fraction decreases linearly. Likewise, with equilibrium swelling, samples resistant to non-polar solvents contain higher amounts of CR. The highest amounts of the leached fraction were obtained in the CR/SBR (50/50 by wt.) sample, with W_q_^T^ = 0.51 mg/mg and W_q_^M^ = 0.17 mg/mg.

**Figure 11:**
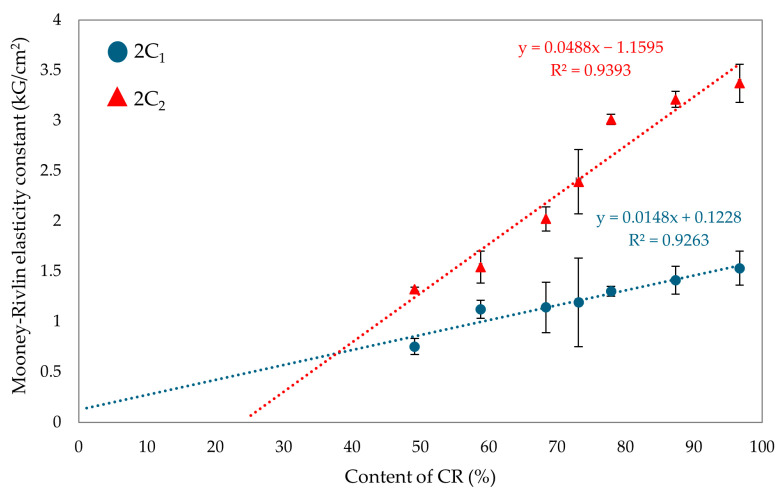
Effects of CR content on Mooney–Rivlin elasticity constants.

The values of the first elasticity constant (2C_1_) are directly proportional to the degree of cross-linking [62,63,64]. The vulcanizate containing only SBR was not tested for the elasticity constant, as the samples would have been destroyed during the test due to the lack of SnO cross-linking. For the CR/SBR (90/10 by wt.) vulcanizate, the 2C_1_ value was 1.41 kG/cm^2^ (Table 2), which was the highest value observed among elastomeric composites. The highest value of the first elastic constant was obtained for the CR vulcanizate (2C_1_ = 1.53 kG/cm^2^). The value of the second elasticity constant (2C_2_) could be interpreted as a measure of the deviation of the formed network from the ideal network. The higher the value of 2C_2_, the greater the deviation [62,63,64]. The highest value of 2C_2_ was obtained for CR (100 by wt.) vulcanizate (2C_2_ = 3.37 kG/cm^2^). However, for the CR/SBR (60/40 by wt.) vulcanizate, the 2C_2_ value was 1.54 kG/cm^2^. The results of the elastic constant measurements indicate that the quantity of elastomers used affects the vulcanizates’ cross-linking degree. Figure 11 illustrates the impact of the CR content on the value of Mooney–Rivlin elasticity constants. The higher the CR content, the more knots and more deviations in the elastomeric network. The cross-linking agent (SnO) reacts with the chloroprene rubber during cross-linking, which creates a spatial interelastomeric network and affects the values of elasticity constants.

**Figure 12:**
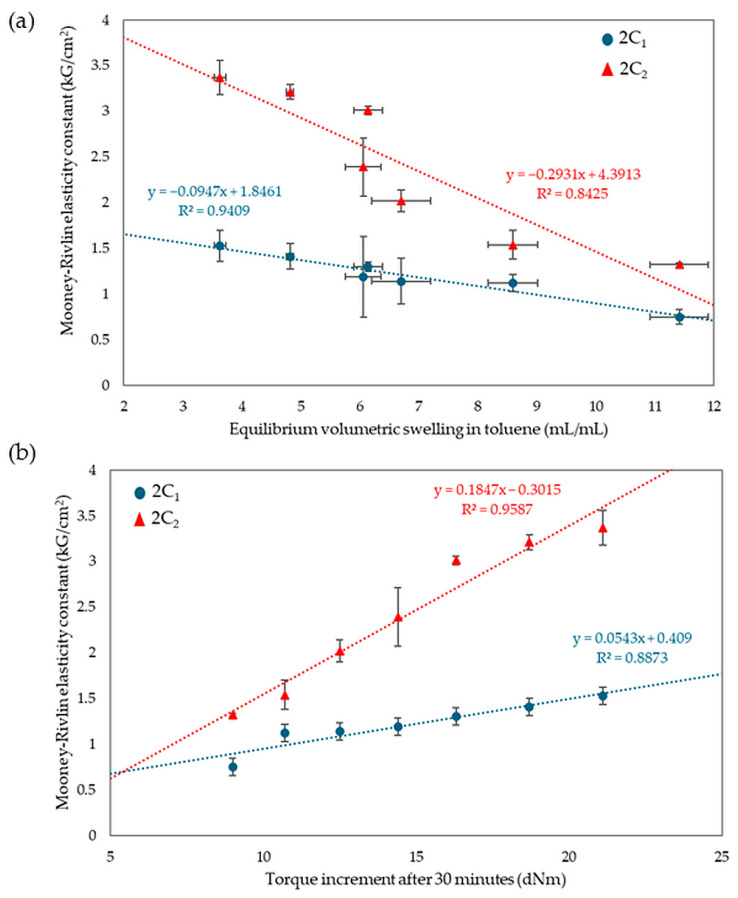
Relationships of equilibrium volumetric swelling in toluene (**a**) and the effect of torque increments with elasticity constants (**b**).

Analyzing the results of equilibrium swelling and constant elasticity and vulcametric properties allows the same conclusions to be drawn. A higher number of cross-bonds is formed in vulcanizates with a higher amount of CR in the CR/SBR composites. This relationship may be due to strong interactions between tin(II) oxide and chloroprene rubber. In contrast, SnO does not react with styrene–butadiene rubber if chloroprene rubber is not used. However, the results do not exclude at least the partial macroscopic miscibility of the elastomers used. During the heating, bonds are formed between rubber macromolecules in the composite, as solvent resistance indicates. Figure 12 shows the relationship between elasticity constants, equilibrium volumetric swelling in toluene, and torque increments after 30 min. Samples with higher ΔT_30_ values assumed higher values of elasticity constants, while for equilibrium swelling the trend was the opposite, with smaller Q_v_^T^ values correlated with higher values of elasticity constants.

**Figure 13:**
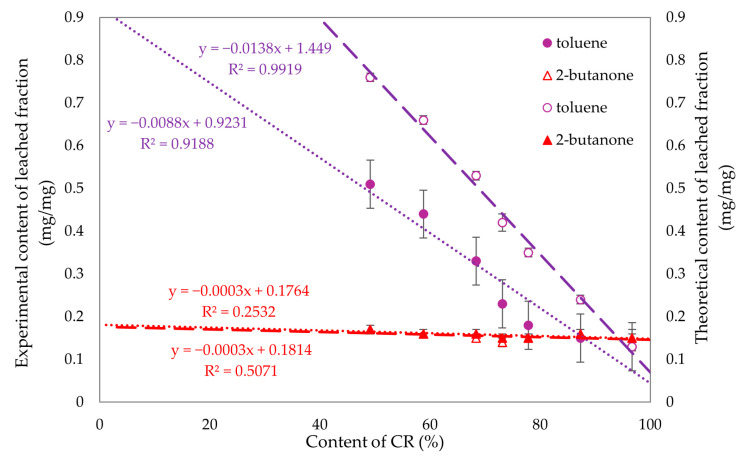
Experimental (filled signs) and theoretical (empty signs) values of leached fractions in toluene and 2-butanone of tested CR/SBR/SnO compositions.

To compare the experimental and theoretical values obtained for the fraction leached in toluene and 2-butanone, Figure 13 was created, enabling us to examine the correlation between chloroprene rubber content and these values. Linear regression was used to fit trend lines. The differences in the fraction leached in toluene were evident, while no significant differences were observed for 2-butanone. For compositions with over 70% chloroprene rubber content, the fraction leached in both toluene and 2-butanone yielded similar values. Therefore, it can be concluded that for compositions above 70% CR, the resistance to these solvents is comparable.

#### 2.3.2. Mechanical Properties of Cured CR/SBR Blends Before and After Thermo-Oxidative Aging

Mechanical properties are some of the basic ones used to describe rubber products. They allow us to define tensile strength, the relative elongation, and also the stress at a given elongation. Based on these parameters, it is possible to determine the suitability of a given product and whether it meets the requirements for various applications. By analyzing tensile strength, engineers and scientists can experiment with various elastomer compositions to enhance mechanical properties, making the material stronger, more durable, and resistant to environmental factors like temperature change or chemical exposure. In summary, tensile strength testing is key to understanding elastomers’ mechanical behavior, ensuring they meet application demands, increasing durability, and improving overall performance. Table 3 illustrates the results of mechanical properties before and after thermo-oxidative aging.

**Figure 14:**
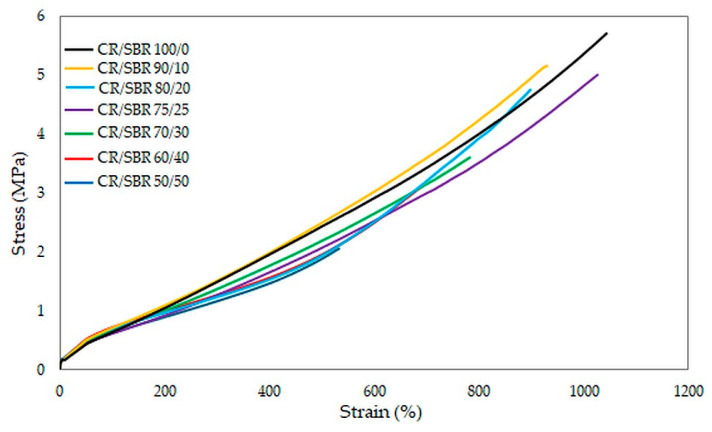
Stress–strain curves of CR/SBR composites cross-linked with tin(II) oxide at T = 433 K; t = 30 min.

The results of the tests unequivocally confirmed that the CR/SBR/SnO vulcanizates are characterized by high deformability, as each of the tested samples reached the maximum range of relative elongation in the measuring device used. The best mechanical properties among cured blends were demonstrated by the CR/SBR (90/10 by wt.) vulcanizate, where tensile strength equaled 5.2 MPa. Other compositions owning more than 75 phr of CR did not differ significantly in tensile strength value (TS_b_ = 4.8–5.0 MPa). Furthermore, most of the samples exceeded more than 800% in elongation. This demonstrates the elastic behavior of the vulcanizates. The values of stresses at given elongations did not differ significantly, and it is noticeable that the higher the content of chloroprene rubber, the higher the value of stress (Figure 14). This phenomenon is related to higher quantities of interelastomeric bonds which depend on the amount of CR because they initiate the cross-linking process through tin(II) oxide.

**Figure 15:**
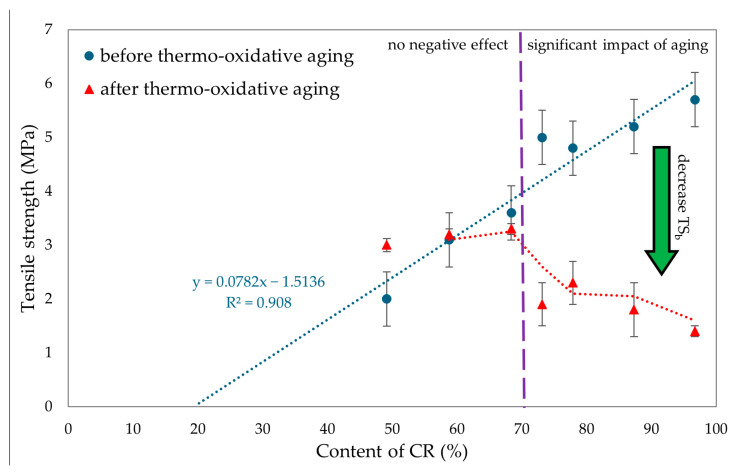
Comparison of tensile strength before and after thermo-oxidative aging for tested CR/SBR/SnO vulcanizates.

**Figure 16:**
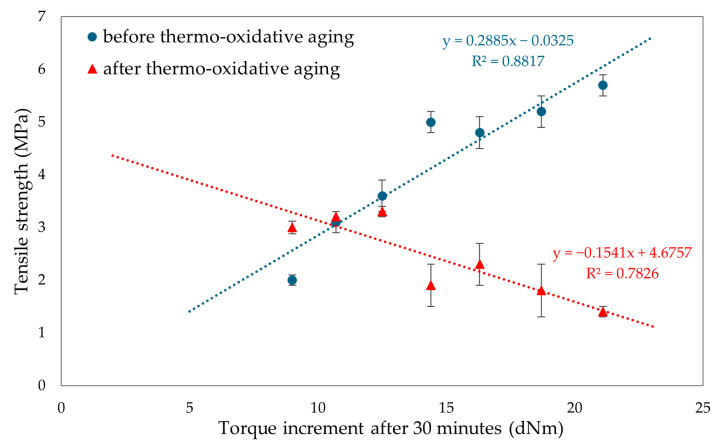
Influence of torque increment on tensile strengths before and after thermo-oxidative aging for tested CR/SBR/SnO vulcanizates.

To evaluate the resistance of CR/BR vulcanizates to thermo-oxidative aging, the samples were kept at 70 °C for 7 days. After aging, all tested composites showed a slight increase in stress with elongation. Hence, it was concluded that thermo-oxidative aging stiffened the tested composites. The best mechanical properties among cured blends were demonstrated by the CR/SBR (90/10 by wt.) vulcanizate, where tensile strength equaled 5.2 MPa. With lower CR content, the tensile strength decreased, which was related to the lower degree of cross-linking of the blends. The data in Figure 15 illustrate that compositions containing more than 70% CR underwent high degrees of thermo-oxidative degradation, which was reflected in their tensile strength after aging. It should be wondered, then, why a marked decrease in mechanical properties was noted here. Indeed, thermo-oxidative aging can lead to re-cross-linking, as a result of which new bonds are formed between polymer chains, making the material harder and more brittle. In addition, chain splitting occurs, in which the polymer chains break, reducing the molecular weight and causing a loss of mechanical properties such as tensile strength and elasticity. From Figure 16, it can be seen that the higher CR content caused a greater increase in torsional moment, resulting in greater cross-linking. Greater cross-linking will result in over-cured chain degradation during aging, which impacts mechanical properties (for example, for the CR/SBR = 60/40 by wt., tensile strength rose from 3.2 MPa to 3.3 MPa). For the CR/SBR (90/10 by wt.) and CR composites, the relative elongation after aging changed, and the samples broke at 652% and 285% elongation, respectively. This suggests that the elasticity of these samples deteriorated after aging. Thermo-oxidative aging studies have shown that the use of tin(II) oxide as a cross-linking agent does not benefit anti-oxidation and anti-aging properties due to the way the valence of tin changes depending on environmental conditions [65]. Figure 15 shows the relationship between CR content and tensile strength and compares tensile strength values before and after aging. The results in terms of the mechanical properties show the same correlation as the study of swelling and vulcametric properties. This indicates the higher the degree of cross-linking, the higher the value of tensile strength, which is shown in Figure 16. This is linked to a better-developed interelastomeric network. The use of SnO as a cross-linking agent was found to produce vulcanizates with worse mechanical properties than when ZnO or Fe_2_O_3_ were used as aprotic Lewis’s acid precursors [44,45].

### 2.4. Effect of Tin(II) Oxide Amount on Cross-Linking and Properties CR/SBR Blend

#### 2.4.1. Evaluation of Tin(II) Oxide Quantity on Vulcametric and Swelling Properties

Due to the toxic effect of zinc oxide on aquatic organisms, there is a search for other compounds that allow the cross-linking of elastomeric compositions but are at the same time less toxic to the environment. For this purpose, the studied CR/SBR/SnO compositions were subjected to analysis to determine how much tin(II) oxide is optimal for all properties, and whether less of this can be added than zinc oxide and similar parameters achieved. Hence, the CR/SBR (75/25 by wt.) blend was chosen, a decision guided by previous results and the desire to select the optimal ratio to determine interelastomeric reactions. Table 4 shows the results relating to vulcametric and swelling properties.

**Figure 17:**
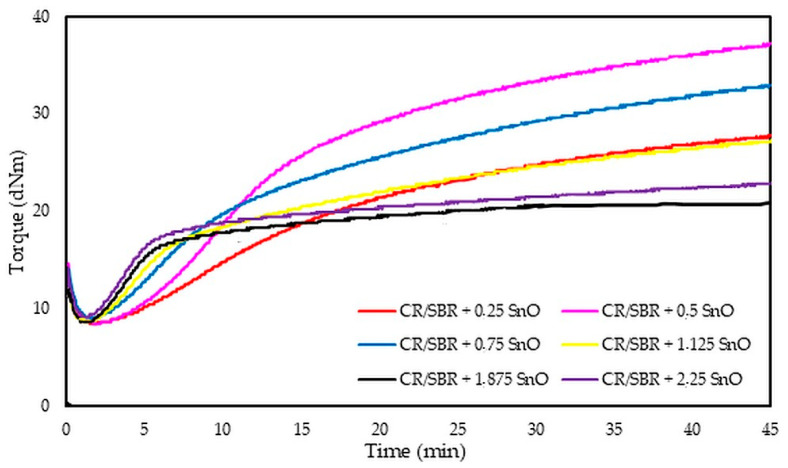
Rheometric curves of CR/SBR (75/25 by wt.) blends cross-linked with different amounts of tin(II) oxide at 160 °C.

The analysis of vulcametric properties shows that the degree of cross-linking of the CR/SBR (75/25 by wt.) blends depends on the SnO content in the system. The cross-linking of the blends occurs without reversion, and the change in torque increment has a marching character, indicating a further progression of cross-linking, like the use of ZnO, Ag_2_O, and Cu_2_O [42,43,44]. The increase in the rheometric torque increment is greatest in a blend containing 0.5 phr. SnO (ΔT_30_ = 24.9 dNm). In contrast, the sample without SnO has the smallest degree of cross-linking (ΔT_30_ = 6 dNm). It should be noted in Figure 17 that the optimal amount of SnO is 0.5 phr. It is worth noting that small amounts of SnO lead to the cross-linking of blends. Moreover, it is important to note that at small amounts of SnO, the CR/SBR composites achieved a significant torque increment, which resulted in a high degree of cross-linking. Particularly noteworthy is the fact that good properties are obtained after the incorporation of small amounts of SnO (0.25–0.75 phr), indicating greater activity in catalyzing the Friedel–Crafts reaction than zinc oxide.

**Figure 18:**
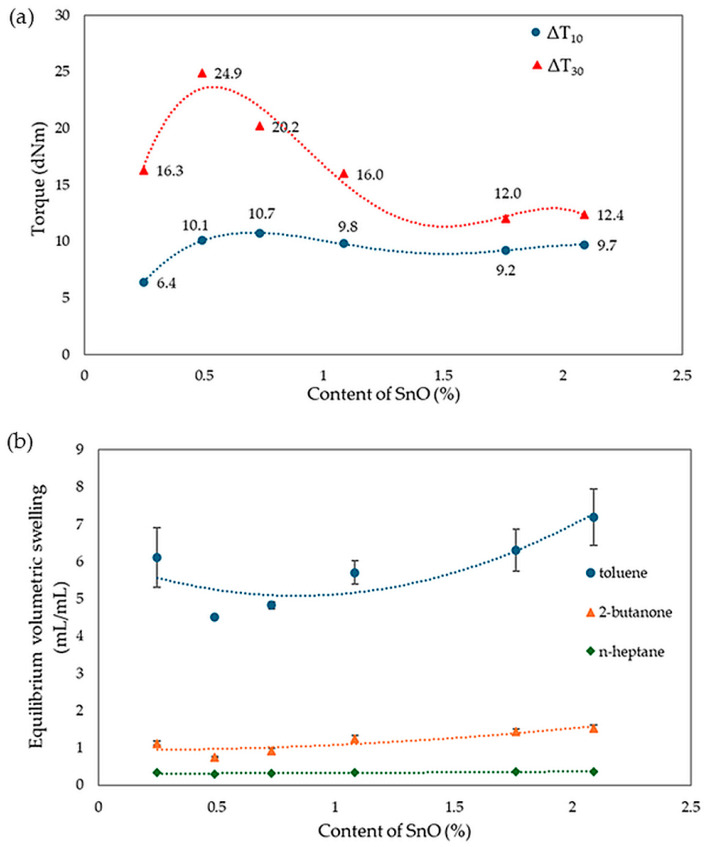
Impact of SnO content on (**a**) torque increment; (**b**) equilibrium swelling in various solvents for tested CR/SBR/SnO composites.

Analyzing the results of equilibrium swelling determinations in selected solvents (Table 4), it was found that the highest degree of cross-linking was seen in the CR/SBR compositions containing 0.5 or 0.75 phr of SnO, because the Q_v_ values for the selected solvents equaled 4.51 mL/mL and 4.83 mL/mL in toluene, respectively. It should be noted that for increasing amounts of tin(II) oxide in the composites tested, the equilibrium swelling value for all solvents increases. This suggests that the higher the amount of SnO, the lower the degree of cross-linking. The differences between the theoretical and experimental values for the content of the fraction leached during swelling, especially the much lower content of the substance extracted by toluene, provide further evidence of the interelastomeric linkage of SBR with CR macromolecules because of the alkylation of phenyl rings with elastomeric polyhalide, catalyzed by SnCl_2_ generated in situ. The same trend can also be observed for Q_v_. The W_q_ values for the sample with 0.5 wt. of SnO showed the highest resistance to solvents (W_q_^T^ = 0.11 mg/mg; W_q_^M^ = 0.10 mg/mg). The remaining samples displayed higher values, indicating a lower degree of cross-linking and less resistance to both polar and non-polar solvents, especially non-polar solvents. The compounds have a higher amount of leaching after exposure to toluene than after exposure to 2-butanone, indicating the less polar nature of the composition. The values of W_q_^T^ for the sample with the content of SnO higher than 0.75 phr increase much more rapidly than for W_q_^M^. This is likely due to the lower number of interelastomeric bonds, which causes unbound SBR to elute upon interaction with toluene. The same correlations were observed in both this study and the previous one. The vulcametric properties are noticeably correlated with the swelling properties. Well-cross-linked samples display high torque increments, higher resistance to solvents, and lower values of equilibrium swelling. Figure 18 presents the effect of cross-linking agent (SnO) content on torque increment and equilibrium swelling in different solvents. It was confirmed by observations that for the CR/SBR (75/25 by wt.) compositions, incorporation at an interval of 0.5–0.75 phr of tin(II) oxide gives optimum swelling and vulcametric properties.

**Figure 19:**
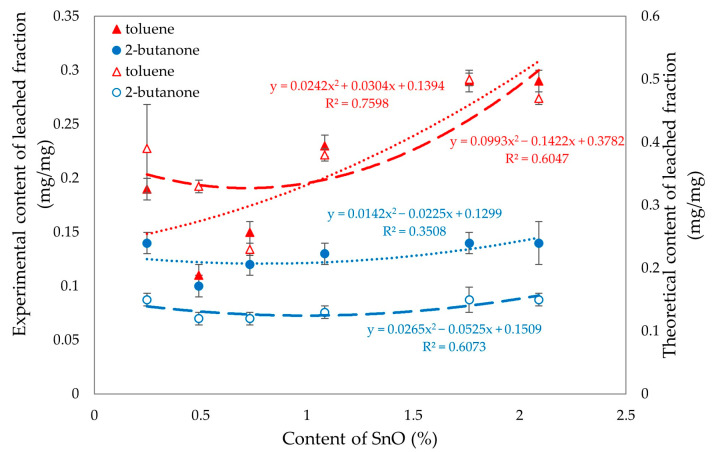
Experimental (filled signs) and theoretical (empty signs) values of leached fractions in toluene and 2-butanone of tested CR/SBR/SnO compositions with various contents of tin(II) oxide.

Figure 19 was created to analyze the differences between the experimental and theoretical values of the eluted fractions in toluene and 2-butanone. The analysis reveals noticeable differences in the values for the fractions eluted in 2-butanone, while no significant differences are observed for toluene. When the SnO content is less than 1%, the fractions eluted in both toluene and 2-butanone yield similar results. This similarity can be attributed to the high degree of cross-linking present in these compositions.

#### 2.4.2. Effect Amount of Tin(II) Oxide on Mechanical Properties of CR/SBR Vulcanizates

The tensile strength of elastomers is strongly related to the degree of cross-linking of their molecular structures. Cross-linking refers to the formation of chemical bonds between polymer chains, creating a network that reinforces the material. As the degree of cross-linking increases, the elastomer becomes more rigid and resistant to deformation, leading to higher tensile strength. The analysis of vulcametric and swelling properties proved that vulcanizates with 0.25–0.75 phr of SnO content showed the highest degree of cross-linking. Tests of mechanical properties confirmed that the sample with the highest degree of cross-linking obtained the greatest tensile strength value. Correlations can be observed between the results of tensile strength and vulcametric and swelling properties.

Analyzing the effect of the amount of SnO incorporated into the CR/SBR (75/25 by wt.) blends on the mechanical properties of their vulcanizates, it was found that the highest tensile strength (TS_b_ = 7.9–8.4 MPa) was achieved with small amounts of SnO (0.25–0.75 phr), while the CR/SBR/SnO (75/25/1.8 by wt.) vulcanizate had a much lower strength (TS_b_ = 5.2 MPa). The CR/SBR (75/25 by wt.) vulcanizates containing 0.25 or 0.5 phr of SnO did not break, even after reaching the maximum elongation range on the measuring device used (>800%). The highest stress value at 100% elongation was obtained for the CR/SBR (75/25/0.25 by wt.) sample, indicating greater resistance to deformation than the other samples and the greater stiffness of the composition (S_e100_ = 1.0 MPa). For the other samples, the S_e100_ value was lower and oscillated in the 0.7–0.5 MPa range. The trend was similar to that demonstrated by the tensile strength values. SnO content between 0.25 and 0.5 phr causes the best mechanical properties. Compared to magnesium and zinc oxides [43], much smaller amounts of tin(II) oxide must be used to achieve satisfactory mechanical properties. These significant differences in the oxide loaded are due to the different strengths of the Lewis acids generated in situ.

**Figure 20:**
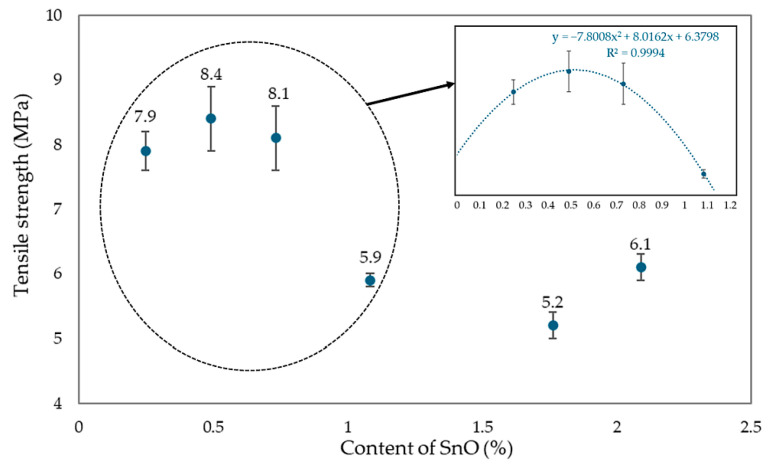
The influence of SnO content in CR/SBR vulcanizates on tensile strength (cross-linking at T = 433 K; t = 30 min).

The mechanical properties of CR/SBR/SnO vulcanizates are related to the degree of cross-linking achieved and changes in the degree of CR crystallinity during the stretching of CR/SBR vulcanizates. Figure 20 compares tensile strength for CR/SBR blends with the addition of SnO from 0 to 2.25 phr. After analysis, it can be seen that CR/SBR vulcanizates with a small amount of SnO (0.25–0.75 phr) obtained a significant difference in tensile strength compared to other samples. It can be assumed that the optimal amount of tin(II) oxide in CR/SBR blends is about 0.5 phr, as confirmed by the analysis.

### 2.5. Flame Resistance of CR/SBR/SnO Vulcanizates

The predominant group of rubber products consists of flammable materials. Consequently, extensive research is currently focused on identifying materials that are thermally stable, flame-resistant, and less likely to ignite. These materials release minimal heat, toxic gasses, and vapors when they burn. Materials are categorized based on their oxygen index (OI) as follows: flammable materials (OI ≤ 21%), flame-retardant materials (OI = 21–28%), and non-combustible materials (OI ≥ 28%) [66]. It is important to note that cured chloroprene rubber is inherently flame-retardant. Flammability was evaluated using the oxygen index.

**Figure 21:**
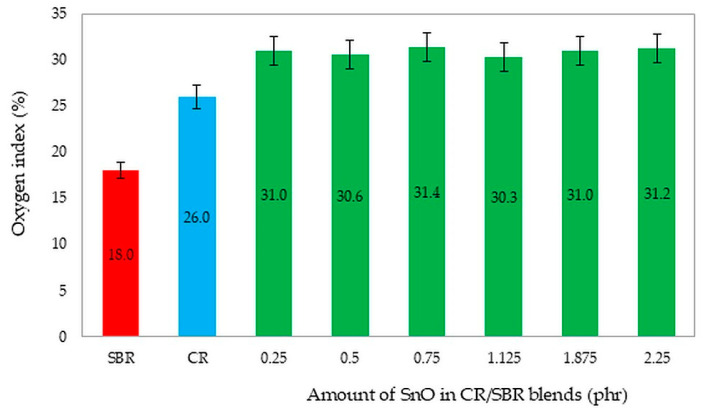
The comparison of the oxygen index values of the tested compositions and pure SBR and CR rubbers. The determination of samples: SBR—styrene–butadiene rubber; CR—chloroprene rubber; CR/SBR = 75/25 by wt.

For the flammability tests, we chose unfilled CR/SBR (75/25 by wt.) vulcanizates containing different amounts of SnO. We selected these compositions to investigate whether the amount of tin(II) oxide could influence flammability. Additionally, the literature values for the oxygen index of pure CR (OI = 26%) [43] and SBR (OI = 18%) [67] are noteworthy. Figure 21 shows a comparison of pure styrene–butadiene and chloroprene rubber, and CR/SBR composites cross-linked with tin(II) oxide. It should be noted that the creation of elastomeric blends and cross-linking with tin(II) oxide improves the non-flammability of the composites. The analysis of oxygen index determinations led to the conclusion that CR/SBR/SnO vulcanizates exhibit good flame resistance and are self-extinguishing [66,67]. However, using SnO as a cross-linking agent resulted in vulcanizates with higher flame resistance (OI = 31%), and the amount of tin(II) oxide did not influence the oxygen index.

All vulcanizates stopped burning after the flame was removed. A lot of smoke was emitted during combustion and the samples lost their elasticity, disintegrating. The addition of SnO caused a “cold fire effect” during combustion and post-combustion delamination of the product. The crucial component of flame-retardant CR/SBR vulcanizates is the addition of tin(II) oxide.

### 2.6. Multivariate Regression Analysis of Selected Parameters of Tested CR/SBR/SnO Composites

Multivariate regression was performed to analyze the various parameter data. Multivariate regression is a powerful statistical tool used to analyze and predict the relationship between a dependent variable and multiple independent variables. Unlike simple linear regression, which examines the influence of a single predictor, multivariate regression considers the simultaneous influence of multiple predictors, offering a more comprehensive understanding of complex systems. In the context of polymer blends, such as chloroprene rubber (CR) and styrene–butadiene rubber (SBR), this approach is particularly valuable for studying how changes in composition and additional factors (e.g., cross-linking agents such as SnO) affect critical properties such as cure characteristics, mechanic behavior, and the degree of cross-linking. Considering multiple variables simultaneously—such as the proportions of CR (%CR–X_1_) and SBR (%SBR–X_2_) in the mix and the content of SnO (%SnO–X_3_)—multivariate regression allows for the quantification of the effect of each component on a specific property (e.g., torque or swelling resistance). Such analysis provides insight into the synergistic and individual contributions of blend components, enabling the precise optimization of material formulations. This chapter discusses the application of multivariate regression to CR/SBR/SnO blends, highlighting its role in correlating blend composition with experimentally measured properties. The regression equations derived in this context serve as predictive models, facilitating the design of blends with tailored performances. The results of the analysis are summarized in Table 5.

**Figure 22:**
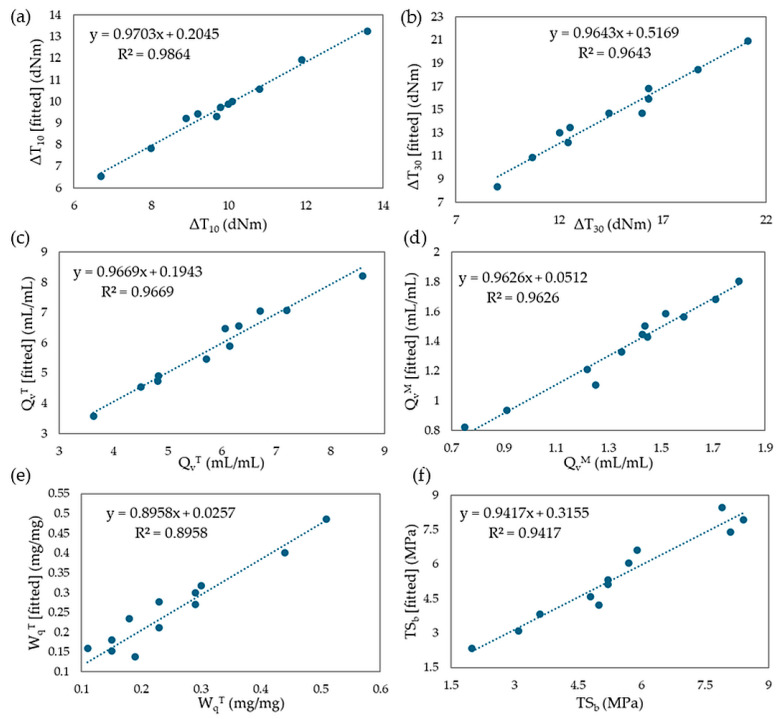
The plots describing fitted values vs. experimental values for (**a**) ΔT_10_; (**b**) ΔT_30_; (**c**) Q_v_^T^; (**d**) Q_v_^M^; (**e**) W_q_^T^; (**f**) TS_b_.

Mathematical regression models were developed based on two rheological properties (ΔT_10_ and ΔT_30_), three swelling properties (Q_v_^T^, Q_v_^M^, W_q_^T^), and one mechanical property (TS_b_). Using experimental values, models were created, and multiple regression equations were formulated. The coefficients β, R^2^ values, and adjusted R^2^ values were calculated. The results of the fitted models were found to be satisfactory, with adjusted R^2^ values ranging from 87.4% to 95.3%. Specifically, the fitted model explains 87.4% of the variation for ΔT_10_ and 94.9% of the variation for ΔT_30_. For the swelling properties, the model for Q_v_^T^ explains 95.3%, Q_v_^M^ explains 94.8%, and W_q_^T^ explains 86.1% of the variation. The fitted model for TS_b_ explains 92.0% of the variation in tensile strength. It can be assumed that the equilibrium swelling in heptane (Q_v_^H^) and the experimental content of the leached fraction of 2-butanone (W_q_^M^) are compositionally independent. Figure 22a–f displays the fit of the models for the selected parameters assessed in this study.

## 3. Materials and Methods

### 3.1. Materials

The following reagents were used to conduct the study: chloroprene rubber (CR) (Baypren^®^216 MV with Mooney Viscosity ML(1 + 4) 100 °C = 38–48 MU from Lanxess GmbH, Dormagen, Germany) and styrene–butadiene rubber (SBR) (KER^®^1502 with Mooney Viscosity ML(1 + 4) 100 °C = 45–55 MU from Synthos S.A., Oswiecim, Poland). These were used as elastomers. Tin(II) oxide (SnO) (Sigma-Aldrich, St. Louis, MO, USA) with a density of 6,4 g/cm^3^ was used as a curing agent, and we used stearic acid with a density of 0.94 g/cm^3^ (Chemical Worldwide Business Sp. z o. o., Slupca, Poland) as a plasticizer.

### 3.2. Method of Testing

CR/BR/SnO blends were prepared conventionally (T = 313–323 K; t = 30 min) using a standard laboratory two-roll mill (model: Laborwalzwerk, Krupp-Gruson, Magdeburg-Buckau, Germany). Then, the blends were pressed in an electrically heated hydraulic press (ZUP, Nysa, Poland) at 433 K for 30 min for curing. Vulcametric measurements were performed using a WG-02 rheometer (ZMCh Metalchem, Gliwice, Poland) at 433 K according to PN-ISO 3417:2004 [68]. The oscillation frequency was 1.67 Hz, and the amplitude of oscillation was fixed at ±3°. By analyzing the kinetics of cross-linking, the minimal rheometric torque (T_M_), the rheometric torque increment after 10 min (ΔT_10_) and 30 min (ΔT_30_), and the scorch time (t_02_) were determined. The degree of cross-linking was determined based on vulcametric measurements, equilibrium swelling in toluene or heptane, extraction with boiling toluene and n-heptane, and the Mooney–Rivlin elasticity constants (2C_1_, 2C_2_). The latter was determined from the Mooney–Rivlin Equation (1):

(1) $$C_{1}+\lambda^{-1}\times C_{2}=\frac{P}{2A_{0}\times (\lambda -\lambda^{-2})}$$

where P is the deformation force at λ (kG), λ is the deformation (λ = l/l_0_), l is the measuring section of the sample loaded with P (cm), l_0_ is the measuring section of the unloaded sample (cm), A_0_ is the cross-sectional area of the unloaded sample (cm^2^), 2C_1_ is the first elastic constant (kG/cm^2^), and 2C_2_ is the second elastic constant (kG/cm^2^).

The network structure and degree of cross-linking CR/BR blends were characterized by FT-IR spectroscopy. FT-IR spectra were made in transmission mode using a Bio-Rad 175C spectrophotometer (Bio-Rad, Herkules, California, USA). The spectra were assessed for the wavenumber range of 3500–600 cm^−1^. Test samples with a 0.03–0.05 mm thickness were prepared using a hydraulic press at 343 K (ZUP, Nysa, Poland).

Mechanical properties were determined using a Zwick machine (Zwick1435/Roell GmbH & Co. KG, Ulm, Germany) conforming to PN-ISO 37:2007: stress at elongations of 100%, 200%, and 300% (S_e100_, S_e200_, S_e300_); tensile strength (TS_b_); and elongation at break (E_b_) [69]. The mechanical parameters were also tested after thermo-oxidative aging at 70 °C for 7 days.

The Fire Testing Technology Apparatus device was used to determine the flammability of vulcanizates using the oxygen index. The test was performed using samples with dimensions of 50 mm × 10 mm × 4 mm. The test was performed at a constant nitrogen flow rate (400 L/h). We selected the oxygen concentration to allow for the complete combustion of the sample within 180 ± 10 s. Flammability in air was determined using the same samples as in the oxygen index method. The sample in a vertical position was ignited with a gas burner for 5 s and its combustion time (t_c_) was measured [70]. The oxygen index (OI) was calculated using Formula (2):

(2) $$OI=\frac{O_{2}}{O_{2}+N_{2}}\cdot 100\%$$

where O_2_ is the oxygen flow rate (l/h) and N_2_ is the nitrogen flow rate (l/h).

An atomic force microscope (AFM, Metrology Series 2000 from Molecular Imaging, Culver City, CA, USA) was used to examine the surface of the vulcanizates. The samples for measurement were vulcanized in a steel mold, with a glass plate placed in the mold to obtain a smooth sample surface. Imaging was performed using a conical (dilation angle < 20°) silica scanning head of about 15–20 μm in height. This operated in oscillation mode at a frequency resonance of around 170 kHz. Image analysis was performed using the WS × M program developed by Horcas et al. [71].

The thermal changes and cross-linking temperature range of the elastomeric blends were investigated using a DSC1 from Mettler Toledo (Mettler-Toledo, Columbus, OH, USA). The thermal properties were measured over a temperature range of −120 to 250 °C, at a heating rate of 10 °C/min, and using liquid nitrogen as a coolant. Before measurement, the DSC analyzer was calibrated using two calibrators: indium and n-octane.

### 3.3. Statistical Analysis

Considering the complexity and the many features that affect the prediction of mechanical, vulcametric, and swelling properties of elastomeric compositions, a model was proposed that estimated selected parameters based on composition. For this purpose, linear regression models were used as it is also of interest to determine the influence of various variables on the results of the model. For this purpose, input data consisting of three factors (CR, SBR, and SnO content) were defined. Before applying any linear regression technique, data analysis was conducted to better understand the data to help to improve model validation. Several analytical methods, such as data preprocessing, outlier detection, and covariance and correlation analysis, were applied to reduce the number of features and remove instances that could reduce the final accuracy. Multivariate regressions were then performed according to Equation (3):

(3) $$Y=\beta_{0}+\beta_{1}\cdot X_{1}+\beta_{2}\cdot X_{2}+\cdots +\beta_{n}\cdot X_{n}$$

where Y is the dependent variable, X_n_ is the independent variable, β_n_ is the coefficients of the multiple regression equation, n is the number of variables, and β_0_ is the intercept.

Then, based on the formula, the adjusted R^2^ values were calculated using Equation (4):

(4) $$\bar{R^{2}}=1-(1-R^{2})\frac{n-1}{n-p-1}$$

where p is the total number of explanatory variables in the model (excluding the intercept), and n is the sample size.

Multivariate regression analysis was performed with Excel using the statistical functions in the Analysis ToolPak plugin at a confidence interval (α) equal to 0.95.

### 3.4. Procedure of Investigation

**Figure 23:**
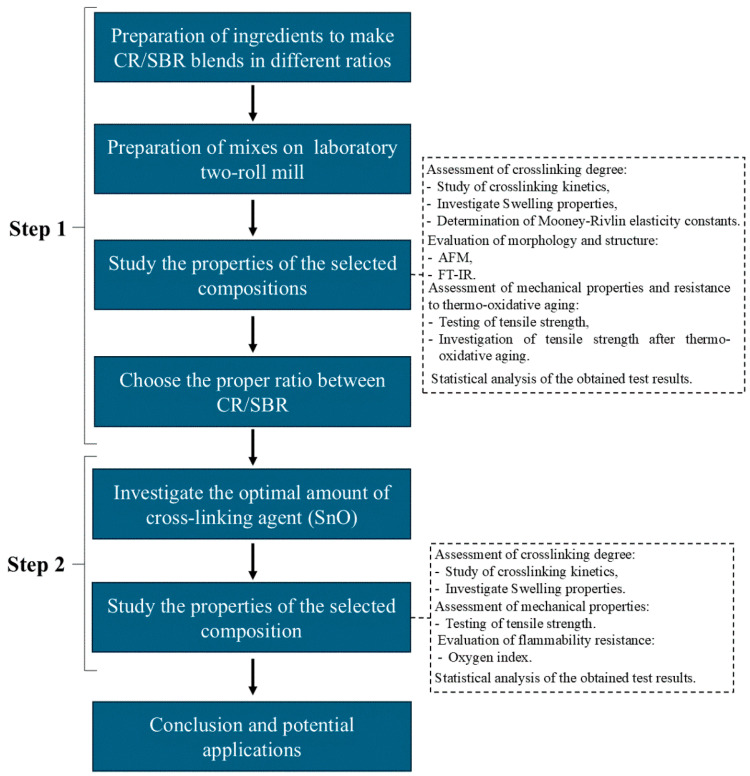
Scheme of testing order.

This study was divided into two stages. The first stage involved selecting the appropriate ratio between styrene–butadiene rubber and chloroprene rubber and examining their properties. In the second stage, the CR/SBR composition with the best properties was selected, and various amounts of tin(II) oxide were incorporated. This approach was used to determine how the amount of cross-linking agent affects the properties of the vulcanizates. Figure 23 illustrates what the procedure for testing elastomeric compositions looked like.

## 4. Conclusions

Tin(II) oxide can be used as an unconventional curing agent for new blends of chloroprene and styrene butadiene rubber (CR/SBR). The cross-linking method we propose allows for the elimination of zinc oxide, which is highly toxic to organisms and causes long-term and adverse changes in the aquatic environment (EU Directive No. 1999/45/EG, 67/548/EEC, 88/379/EEC) [72]. In addition, the toxicity and carcinogenicity of thiourea derivatives determine the need to eliminate ethylene thiourea from rubber composites, which is commonly used as an accelerator during the cross-linking of chloroprene rubber and its mixtures using ZnO/MgO. We achieved these goals in our work.

The degree of cross-linking of the CR/SBR vulcanizates and their strength properties improve with increasing CR content in the blends. The best properties were obtained for CR/SBR vulcanizates with CR contents higher than 75 phr. Compared with zinc oxide-cross-linked vulcanizates, smaller amounts of tin(II) oxide allow similar properties to be achieved. Adding 0.25–0.75 phr of tin(II) oxide to the CR/SBR composites results in the best mechanical properties and a high degree of cross-linking.

During the cross-linking of chloroprene and styrene–butadiene rubber blends with SnO, the alkylation of phenyl rings by an elastomeric polyhalide, catalyzed by Lewis’s acid generated in situ, occurs. During this reaction, the donor of the metal ions is tin(II) oxide, and the acceptor of these ions is CR rubber. The analysis of the spectra in FT-IR confirmed the cross-linking of CR/SBR blends occurs because of interelastomer alkylation by the Friedel–Crafts reaction of SBR phenyl rings with the elastomeric polyhalide and the autoalkylation of these rings by SBR chains containing vinyl side groups. AFM analysis determined the changes in morphology from a two-phase system to droplet-dispersed SBR particles of micrometric size in the CR continuous phase.

The results of the flammability test using the oxygen index provided information showing that all CR/SBR compositions tested are non-flammable. All CR/SBR vulcanizates are characterized by good flame resistance (OI = 31%) and can be classified as self-extinguishing materials. The presence of flame retardants in the test composites is not required to obtain satisfactory fire resistance parameters.

The previously unknown elastomeric compositions obtained in the work can be used to produce rubber products with a wide range of applications. The proposed technology can be successfully implemented by plastics industry plants on an industrial scale on existing production lines, without the need to modify the machinery. This technology will bring its users tangible benefits related to ecological considerations and work safety resulting from the elimination of hazardous and harmful cross-linking substances. The most important advantage of cross-linked elastomeric composites in which interelastomer reactions occur is their limited flammability, which is of great importance for most rubber products, which are flammable materials. Potential applications of the produced elastomeric materials include oil-resistant products, chemical-resistant coatings, heat-resistant conveyor belts, flame-retardant plates, fire-resistant seals, hoses, electrical wire insulation, insulating roof membranes, or non-flammable protective clothing elements for the fire brigade, police, and army.

## Figures and Tables

**Table 1:** Differential scanning calorimetry (DSC) results of CR/SBR blends cross-linked with tin(II) oxide.

CR/SBR (phr)	T_g CR_	T_g SBR_	T_onset_	T_endset_	ΔH
	°C	°C	°C	°C	J/g
50/50	−36.53	−49.70	140	243	2.66
60/40	−37.65	−50.12	138	188	1.78
70/30	−37.24	−49.84	132	199	3.36
75/25	−38.03	−49.96	130	154	5.79
80/20	−38.06		152	196	4.56
90/10	−35.95		125	198	3.74
100/0	−37.62	-	169	181	2.98

T_g SBR_—glass-transition temperature of SBR; T_g CR_—glass-transition temperature of CR; T_onset_—the onset temperature of cross-linking; T_endset_—the endset temperature of cross-linking; ∆H—enthalpy of cross-linking.

**Table 2:** Composition and selected parameters characterizing the cross-linking of CR/SBR/SnO blends and the properties of their vulcanizates (cross-linked at T = 433 K; t = 30 min).

**Compositions of blends (phr)**								
CR	0	50	60	70	75	80	90	100
SBR	100	50	40	30	25	20	10	0
Stearic acid	—	0.5	0.6	0.7	0.75	0.8	0.9	1
SnO	—	1.20	1.44	1.68	1.80	1.92	2.16	2.40
**Vulcametric properties of blends**								
t_02_ (min)	1.9	2.5	2.0	2.1	1.9	1.8	1.6	1.9
T_M_ (dNm)	13.0	10.2	9.4	9.1	9.3	9.0	9.3	9.9
ΔT_10_ (dNm)	1.3	6.7	8.0	8.9	10.0	10.8	11.9	13.6
ΔT_30_ (dNm)	1.5	9.0	10.7	12.5	14.4	16.3	18.7	21.1
** Swelling properties of vulcanizates **								
Q_v_^T^ (mL/mL)	D	11.41 ± 0.49	8.59 ± 0.42	6.70 ± 0.50	6.06 ± 0.30	6.14 ± 0.24	4.82 ± 0.06	3.63 ± 0.10
Q_v_^M^ (mL/mL)	D	1.80 ± 0.03	1.71 ± 0.01	1.59 ± 0.06	1.44 ± 0.03	1.43 ± 0.04	1.35 ± 0.03	1.22 ± 0.03
Q_v_^H^ (mL/mL)	D	0.46 ± 0.07	0.56 ± 0.01	0.40 ± 0.01	0.33 ± 0.01	0.29 ± 0.01	0.20 ± 0.01	0.50 ± 0.01
W_q_^T^ (mg/mg)	D	0.51 ± 0.01	0.44 ± 0.01	0.33 ± 0.01	0.23 ± 0.02	0.18 ± 0.01	0.15 ± 0.01	0.13 ± 0.01
W_q_^M^ (mg/mg)	D	0.17 ± 0.01	0.16 ± 0.01	0.15 ± 0.01	0.14 ± 0.02	0.15 ± 0.01	0.16 ± 0.01	0.15 ± 0.01
W_qt_^T^ (mg/mg)	D	0.76 ± 0.01	0.66 ± 0.01	0.53 ± 0.01	0.42 ± 0.01	0.35 ± 0.01	0.24 ± 0.01	0.13 ± 0.01
W_qt_^M^ (mg/mg)	D	0.17 ± 0.01	0.16 ± 0.01	0.16 ± 0.01	0.15 ± 0.01	0.15 ± 0.01	0.16 ± 0.01	0.15 ± 0.02
** Elasticity constants of vulcanizates **								
2C_1_ (kG/cm^2^)	—	0.75 ± 0.08	1.12 ± 0.09	1.14 ± 0.25	1.19 ± 0.44	1.30 ± 0.05	1.41 ± 0.14	1.53 ± 0.17
2C_2_ (kG/cm^2^)	—	1.32 ± 0.02	1.54 ± 0.16	2.02 ± 0.12	2.39 ± 0.32	3.01 ± 0.05	3.21 ± 0.08	3.37 ± 0.19

CR—chloroprene rubber; SBR—styrene–butadiene rubber; SnO—tin(II) oxide; t_02_—scorch time; T_M—_minimum torque; ΔT_10_/ΔT_30_—torque increment after 10 or 30 min of heating; Q_v_—equilibrium volumetric swelling in toluene (Q_v_^T^), n-heptane (Q_v_^H^), or 2-butanone (Q_v_^M^), respectively; W_q_—experimental content of the fraction leached during swelling, respectively, in toluene (W_q_^T^) or 2-butanone (W_q_^M^); W_qt_—theoretical content of the fraction leached during swelling in toluene (W_qt_^T^) or 2-butanone (W_qt_^M^); respectively; 2C_1_/2C_2_—Mooney–Rivlin elasticity constants; D—the sample has dissolved.

**Table 3:** The effect of the amount of CR in CR/SBR/SnO compositions cross-linked at T = 433 K/t = 30 min on selected parameters used to characterize mechanical properties before and after thermo-oxidative aging (T = 343 K; t = 7 days).

Amount of CR (phr)	50	60	70	75	80	90	100
**Mechanical properties before thermo-oxidative aging**							
S_e100_ (MPa)	0.4 ± 0.1	0.4 ± 0.1	0.4 ± 0.1	0.5 ± 0.1	0.5 ± 0.1	0.5 ± 0.1	0.5 ± 0.1
S_e200_ (MPa)	0.5 ± 0.1	0.5 ± 0.1	0.5 ± 0.1	0.6 ± 0.1	0.6 ± 0.1	0.6 ± 0.1	0.7 ± 0.1
S_e300_ (MPa)	0.5 ± 0.1	0.6 ± 0.1	0.6 ± 0.1	0.8 ± 0.1	0.8 ± 0.1	0.7 ± 0.1	0.8 ± 0.1
TS_b_ (MPa)	2.0 ± 0.1	3.1 ± 0.2	3.6 ± 0.3	5.0 ± 0.2	4.8 ± 0.3	5.2 ± 0.3	5.7 ± 0.2
E_b_ (%)	540	690	>800	>800	>800	>800	>800
**Mechanical properties after thermo-oxidative aging (T = 343 K; t = 7 days)**							
S_e100′_ (MPa)	0.4 ± 0.1	0.5 ± 0.1	0.5 ± 0.1	0.5 ± 0.1	0.5 ± 0.1	0.5 ± 0.1	0.7 ± 0.1
S_e200′_ (MPa)	0.5 ± 0.1	0.6 ± 0.1	0.6 ± 0.1	0.7 ± 0.1	0.7 ± 0.1	0.7 ± 0.1	1.1 ± 0.1
S_e300′_ (MPa)	0.6 ± 0.1	0.8 ± 0.1	0.7 ± 0.1	0.9 ± 0.1	0.8 ± 0.1	0.8 ± 0.1	1.4 ± 0.1
TS_b_’ (MPa)	3.0 ± 0.2	3.2 ± 0.1	3.3 ± 0.1	1.9 ± 0.4	2.3 ± 0.4	1.8 ± 0.5	1.4 ± 0.1
E_b_’ (%)	460	580	>800	703 ± 91	>800	652 ± 98	285 ± 17

S_e100_, S_e200_, S_e300_—stress at 100, 200, or 300% relative elongation before thermo-oxidative aging; TS_b_—tensile strength before thermo-oxidative aging; E_b_—relative elongation at break before thermo-oxidative aging; S_e100′_, S_e200′_, S_e300′_—stress at 100, 200, or 300% relative elongation after thermo-oxidative aging; TS_b_’—tensile strength after thermo-oxidative aging; E_b_’—relative elongation at break after thermo-oxidative aging.

**Table 4:** The composition and selected parameters characterizing the effect of the amount of SnO on the cross-linking of CR/SBR (75/25 by wt.) blends and the properties of their vulcanizates (cross-linking at T = 433 K; t = 30 min).

Composites CR/SBR = 75/25 by wt.						
Amount of SnO (phr)	0.25	0.50	0.75	1.125	1.875	2.25
**Vulcamteric properties of blends**						
t_02_ (min)	1.9	2.1	1.7	1.5	1.6	1.4
T_M_ (dNm)	8.4	8.4	9.0	8.6	8.6	9.1
ΔM_10_ (dNm)	6.4	10.1	10.7	9.8	9.2	9.7
ΔM_30_ (dNm)	16.3	24.9	20.2	16.0	12.0	12.4
**Swelling properties of vulcanizates**						
Q_v_^T^ (mL/mL)	6.11 ± 0.80	4.51 ± 0.03	4.83 ± 0.10	5.71 ± 0.31	6.31 ± 0.56	7.20 ± 0.76
Q_v_^M^ (mL/mL)	1.12 ± 0.05	0.75 ± 0.02	0.91 ± 0.07	1.25 ± 0.08	1.45 ± 0.06	1.52 ± 0.09
Q_v_^H^ (mL/mL)	0.33 ± 0.01	0.29 ± 0.01	0.31 ± 0.02	0.34 ± 0.01	0.36 ± 0.01	0.36 ± 0.01
W_q_^T^ (mg/mg)	0.19 ± 0.01	0.11 ± 0.01	0.15 ± 0.01	0.23 ± 0.01	0.29 ± 0.01	0.29 ± 0.01
W_q_^M^ (mg/mg)	0.14 ± 0.01	0.10 ± 0.01	0.12 ± 0.01	0.13 ± 0.01	0.14 ± 0.01	0.14 ± 0.02
W_qt_^T^ (mg/mg)	0.39 ± 0.07	0.33 ± 0.01	0.23 ± 0.01	0.38 ± 0.01	0.50 ± 0.01	0.47 ± 0.01
W_qt_^M^ (mg/mg)	0.15 ± 0.01	0.12 ± 0.01	0.12 ± 0.01	0.13 ± 0.01	0.15 ± 0.02	0.15 ± 0.01

For descriptions, see Table 2.

**Table 5:** Parameters of multivariable regression.

Parameter	Equation Coefficients				R^2^	Adjusted R^2^
ΔT_10_	β_0_	β_1_	β_2_	β_3_	0.999	0.874
	−2.6434	0.1645	0.0207	0.0593		
ΔT_30_	β_0_	β_1_	β_2_	β_3_	0.964	0.949
	−30.7765	0.5562	0.2607	−0.8900		
Q_v_^T^	β_0_	β_1_	β_2_	β_3_	0.967	0.953
	5.9670	−0.0610	0.0945	1.5160		
Q_v_^M^	β_0_	β_1_	β_2_	β_3_	0.963	0.948
	−1.6750	0.0165	0.0409	0.5545		
W_q_^T^	β_0_	β_1_	β_2_	β_3_	0.896	0.861
	−0.0619	−0.0009	0.0097	0.0937		
TS_b_	β_0_	β_1_	β_2_	β_3_	0.942	0.920
	35.4285	−0.2291	−0.3694	−3.1118		

## Data Availability

The original contributions presented in the study are included in the article, further inquiries can be directed to the corresponding author.
